# Design of Anion Exchange Membranes and Electrodialysis Studies for Water Desalination

**DOI:** 10.3390/ma9050365

**Published:** 2016-05-12

**Authors:** Muhammad Imran Khan, Rafael Luque, Shahbaz Akhtar, Aqeela Shaheen, Ashfaq Mehmood, Sidra Idress, Saeed Ahmad Buzdar, Aziz ur Rehman

**Affiliations:** 1 Department of Chemistry, The Islamia University of Bahawalpur, Bahawalpur 63100, Pakistan; raoimranishaq@gmail.com (M.I.K.); akhtar.shahbaz15@gmail.com (S.A.); aqeelashaheen1@gmail.com (A.S.); ksidra259@gmail.com (S.I.); 2 Departamento de Universidad de Córdoba, Edificio Marie Curie, Ctra Nnal IV-A, Km396, Córdoba E14014, Spain; q62alsor@uco.es; 3 Department of Chemistry, Bahaudin Zakariya University Multan, Multan 60800, Pakistan; amqureshi@edu.com.pk; 4 Department of Physics, The Islamia University of Bahawalpur, Bahawalpur 63100, Pakistan; saeed.buzdar@iub.edu.pk; 5 School of Chemistry and Material Science, University of Science and Technology of China, Hefei 230026, China

**Keywords:** anion exchange membrane, BPPO, methyl(diphenyl)phosphine, electrodialysis, NaCl

## Abstract

Anion exchange membranes are highly versatile and nowadays have many applications, ranging from water treatment to sensing materials. The preparation of anion exchange membranes (AEMs) from brominated poly(2,6-dimethyl-1,6-phenylene oxide) (BPPO) and methyl(diphenyl)phosphine (MDPP) for electrodialysis was performed. The physiochemical properties and electrochemical performance of fabricated membranes can be measured by changing MDPP contents in the membrane matrix. The influence of a quaternary phosphonium group associated with the removal of NaCl from water is discussed. The prepared membranes have ion exchange capacities (IEC) 1.09–1.52 mmol/g, water uptake (W_R_) 17.14%–21.77%, linear expansion ratio (LER) 7.96%–11.86%, tensile strength (TS) 16.66–23.97 MPa and elongation at break (*E*_b_) 485.57%–647.98%. The prepared anion exchange membranes were employed for the electrodialytic removal of 0.1 M NaCl aqueous solution at a constant applied voltage. It is found that the reported membranes could be the promising candidate for NaCl removal via electrodialysis.

## 1. Introduction

Ion exchange membranes (IEM) have been extensively investigated in the past century [1], attracting both commercial and academic interests in water treatment and energy conversion processes [2]. For instance, they can be employed in electrodialysis (ED) to concentrate or deionize aqueous electrolyte solutions [3,4] and in diffusion dialysis to recover acid or alkali from waste acid or alkali solutions [5]. They can also detach acidic gas via carrier transport [6,7,8], act as solid electrolytes in fuel cells [9,10] and behave as sensing materials in sensors [11]. Among these applications, the separation of specific salts from aqueous electrolyte solutions through ED is one of the most crucial utilizations as far as environmental protection is concerned [12]. Many types of polymers have been used in AEM fabrication, for instance, polystyrene [13,14,15], polysulfone [16], polyether imide [17], poly(2,6-dimethyl-1,6-phenylene oxide) (PPO) [18] poly(arylene ether) [19], poly(phthalazinone ether ketone) (PPEK) [20] and poly(phthalazinone ether sulfone ketone) (PPESK) [21]. These have been subjected to chloromethylation and thereafter converted for distinct membrane utilization such as in fuel cells [22], biomedical devices [23,24], evaporation [25], solid state polypeptide synthesis, *etc.* [26]. Chloromethyl ether (CME) was employed commonly for chloromethylation process. However, CME is carcinogenic and hazardous, hence its use has been restricted since 1970 [27]. Alternative techniques have been suggested to minimize the hazardous impacts of AEMs as *in situ* generation [28], chlorination of vinyl aromatic polymers using sulfuryl chloride [29] and polymerization of halomethyl substituted aromatic monomers such as chloro methyl styrene [30].

A facile approach for the synthesis of AEMs is benzylic bromination of polymers, resulting in formation of quaternary ammonium moieties. Polymers such as polysulfone [31,32], PPO [33,34], poly(phenylene) [35], and poly(arylene ether ketone) [36] have been used to attain such AEMs via bromination and subsequent quaternization. These membranes possess high IEC, ionic conductivity, and low water uptake; hence, they are frequently being used in fuel cell preparation [37,38]. However, they cannot be used for the fabrication of intermolecular AEMs with immense durability and diverse applications.

The aim of the present work is to fabricate anion exchange membranes via brominated poly(2,6-dimethyl-1,4-phenylene oxide) (BPPO) by varying MDPP contents and their characterization by physiochemical and electrochemical methods. Moreover, these membranes were investigated for electrodialytic applications and compared with commercial anion exchange membrane *i.e.*, Neosepta, AMX (Astom corporation, Tokyo, Japan), which is commonly employed for the removal of salt via electrodialysis.

## 2. Experimental Section

### 2.1. Materials

Brominated poly(2,6-dimethyl-1,4-phenyleneoxide) (BPPO) was supplied by Tianwei Membrane Co. Ltd., Weifang, China. Commercial anion exchange membrane Neosepta AMX and cation exchange membrane Neosepta CMX were purchased from ASTOM, Tokyo, Japan. Methyl(diphenyl)phosphine (MDPP) was obtained from Sinopharm Chemical reagent Co. Ltd., Shanghai, China. All other reagents used in the experiments were of analytical grade and commercially available from domestic chemical reagents companies. These reagents were used without further purification. Deionized water (DI water) was used throughout the experiments.

### 2.2. Membrane Preparation

**Scheme 1:**
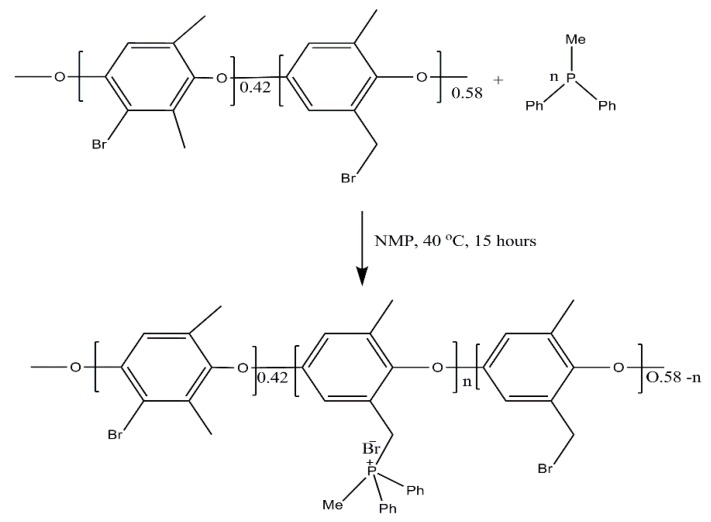
The fabrication of BPPO based anion exchange membranes.

The anion exchange membranes reported here were prepared by the solution-casting method. The casting solution consists of 8% of brominated poly(2,6-dimethyl-1,4-phenylene oxide) (BPPO) in N-Methyl-2-pyrrolidone (NMP) at room temperature. Anion exchange membranes (MDPP-29 to MDPP-43) were fabricated by adding different quantities of methyl(diphenyl)pholsphine (MDPP) into the casting solution to get different properties. The casting solution was stirred vigorously for about 16 h to accelerate the reaction between BPPO and MDPP (methyl(diphenyl)pholsphine). Finally, the solution was casted onto a glass plate and heated at 60 °C (solvent evaporation) for 24 h. The anion exchange membranes were peeled off glass plates and washed with deionized water before characterization and study. Chemical structures for the prepared BPPO based membrane are also represented in Scheme 1.

### 2.3. Characterizations

#### 2.3.1. FTIR Spectra and Thermal Stability

Fourier Transform Infrared (FTIR) spectra of dried membranes were recorded by using the technique attenuated total reflectance (ATR) with FTIR spectrometer (Vector 22, Bruker, Chorley Lancashire, UK) having a resolution of 2 cm^−1^ and a total spectral range of 4000–400 cm^−1^. TGA for the prepared membranes was carried out using a Shimadzu TGA-50H analyser (Shimadzu Corporation, Kyoto, Japan), within the temperature range of 25 °C to 700 °C under nitrogen flow, with a heating rate of 10 °C/min.

#### 2.3.2. Ion Exchange Capacity

IEC for the prepared membranes were measured by the classical Mohr method [39]. To begin with, the membrane samples were equilibrated in 1.0 (M) NaCl solution for two days such that all charge sites were converted into the Cl^−^ form. Then, the membranes were washed carefully with deionized water in order to remove excess amount of NaCl. The washed membranes were then equilibrated with 0.5 (M) Na_2_SO_4_ solutions for two days. The amount of Cl^−^ ions liberated was estimated by titration with 0.05 (M) AgNO_3_ using K_2_CrO_4_ as an indicator. The ion-exchange capacity (IEC; mmol/g) of the membrane was calculated by the equation IEC = *VC*/*m* where *m*, *V* and *C* represents the dry weight of the membrane, titre value during titration and the concentration of AgNO_3_ solution, respectively.

#### 2.3.3. Microscopic Characterizations for AEMs

Membrane morphological characterization was successfully done through a field emission scanning electron microscope (FE-SEM, Sirion200, FEI Company, Hillsboro, OR, USA). Surface and cross-sectional views of membranes were taken from dry membranes. The SEM images of different porous BPPO-based membranes were shown as representative cases.

#### 2.3.4. Water Uptake and Linear Expansion Ratio and Fixed Group Concentration

Hydrophilic nature of membrane was investigated by water uptake (W_R_) measurement. Membrane samples were oven dried and accurately weighed. Then, membranes were immersed in water for 72 h at 25 °C. The weight was again recorded. From the difference in mass before and after the complete drying of the membranes, W_R_ values were calculated as the relative weight gain per gram of the dry sample using the following Equation (1):

(1) $$W_{R}=\left(\frac{W_{\text{WET}}-W_{\text{DRY}}}{W_{\text{DRY}}}\right)\times 100,$$

where W_WET_ and W_DRY_ are the weights of the wet and dry membranes, respectively.

Linear expansion ratio (LER) was calculated at 25 °C water, and has given in Equation (2). All of the membranes were cut into a 2 × 2 cm^2^ piece for the experiment [40]:

(2) $$\text{LER}=\left(\frac{L_{\text{WET}}-L_{\text{DRY}}}{L_{\text{DRY}}}\right)\times 100,$$

where *L*_WET_ and *L*_DRY_ are the lengths of wet and dry membranes, respectively.

#### 2.3.5. Mechanical Property

Tensile strength of dry and hydrated membranes was measured using Q800 dynamic mechanical analyzer (DMA, TA Instruments, NETZSCH, Selb, Bavaria, Germany) at a stretch rate of 0.5 N/min.

#### 2.3.6. Membrane Area Resistance

Membrane area resistance was measured by commercial cell-assembly (MEIEMP-I, Hefei Chemjoy Polymer Material Co., Ltd., Hefei, China) under a constant current mode [41]. It consists of five compartments: two intermediate compartments equipped with two reference electrodes and one quadrate clip for membrane. Particularly, two pieces of Nafion-117 membrane are assembled between electrode and intermediate chambers, to eliminate the influence of electrode reaction. Two intermediate chambers are separated by the membrane clip and tips of the reference electrodes are kept closer to the center of membrane. During the measurement, Na_2_SO_4_ solution (0.5 mol/L) was fed to electrode chambers and NaCl (0.5 mol/L) is fed to the intermediate chambers. This concentration of Na_2_SO_4_ and NaCl is found to be very suitable for measuring the area resistance of membranes. The higher concentration is not sufficient because it results in increased resistance due to the competition among the ions of concentrated solutions on the membrane-exposed region. A constant current is supplied by a direct current power supply (SHEKONIC, Yangzhou Shuanghong Co., Ltd., Yangzhou, China) and the potential between electrodes is read by digital multimeter (model: GDM 8145), Good will instrument Co. Ltd., New Taipei City, Taiwan). Membrane area resistance was calculated according to the values of current, potential and membrane area (7.07 cm^2^).

#### 2.3.7. Membrane Transport Number

Membrane transport number was determined by measuring membrane potential. A cell made of Perspex sheet (Ray Chung Acrylic Enterprise, Kaohsiung, Taiwan), was used for membrane potential measurement, and it had two compartments separated by a membrane circular in shape and having an area of 7.0 cm^2^. The membrane potential was measured by keeping the ratio of salt concentrations of the higher (0.05 M NaCl) to lower (0.01 M NaCl) side constant at 5.0 The potential difference (*E*_m_) across the cell was measured using a multimeter (VC890C+, Shenzhen Victor Hi-Tech. Ltd., Shenzhen, China) connected to Ag/AgCl reference electrodes which were responsible for up to 0.10 mV. The transport number *t*_i_’ was then calculated using the following modified Nernst Equation [42]:

(3) $$E_{m}=\frac{RT}{nF}\left(2t_{i}{}^{'}-1\right)\ln(\frac{a_{1}}{a_{2}}),$$

where *R* is universal gas constant (8.314 J/K·mol), *F* is the Faraday constant (96,487 C/mol), *T* is the absolute temperature (K), *a*_1_ and *a*_2_ are the mean activities of electrolyte solutions and *n* is the electrovalence of counter-ion (*n*_i_ = 1 in this case).

#### 2.3.8. Electrodialysis Stack

**Figure 1:**
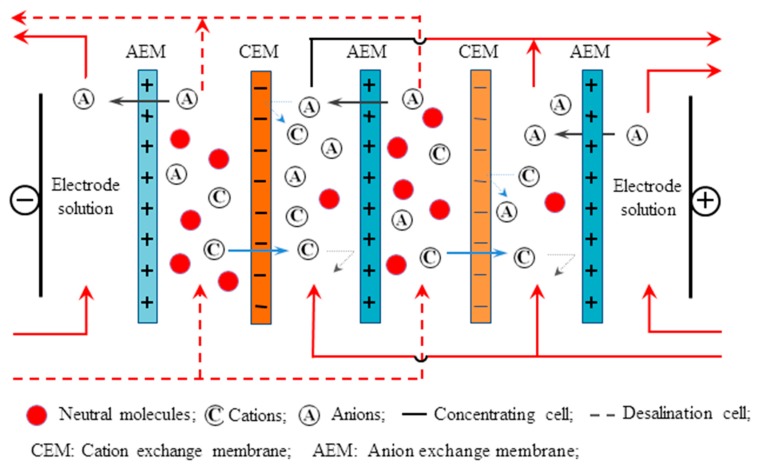
The schematic diagram of an ED stack.

The electrodialysis stack used in this work is represented in Figure 1. It is composed of an anode and a cathode of stainless steel sheets coated with platinum and six electrode cells separated by two CEMs (Neosepta CMX) and three AEMs. The effective area of membrane is 7.0 cm^2^. Anion exchange membranes (MDPP-36 & MDPP-43) and commercial membrane AMX were used in the experiment. A feed solution of (O.1 M NaCl) was pumped in the dilute chamber with a constant flow rate of 60 mL/min. Meanwhile, two electrode cells were fed by 0.3 M Na_2_SO_4_ solutions and connected together to prevent pH change. Before the experiment, each cell was circulated for 30 min to remove the visible bubbles. In the last experiment, the cells were operated at a constant current of value 28 mA/cm^2^. The change in conductivity of an NaCl solution in diluted cells and potential over the stack were recorded after every 5 min.

In an ED stack, the dilute feed stream, concentrate stream, and electrode stream are allowed to flow through the appropriate cell compartment formed by the IEMs. Under the influence of an electrical potential, the negatively charged ions (e.g., chloride) in the dilute stream migrated toward the positively charged anode. These ions pass through the positively charged AEM but are prevented from further migration toward the anode by the negatively charged CEM, and therefore persisted in the concentrated stream, which became concentrated with anions. The positively charged species (e.g., sodium) in the dilute stream migrated and passed through the negatively charged cathode *i.e.*, CEM. These cations also remained in the stream, prevented from further migration toward the cathode by positively charged AEMs. As a result of the anion and cation migration, an electric current flow was observed. An equal number of anions and cations were transferred from the dilute stream into the concentrated stream; hence, the charge balance is maintained in each stream. The overall result of the ED process is an increase in ion concentration in the concentrate stream with a depletion of ions from the dilute solution feed.

This stream may consist of the same composition as the feed stream (e.g., sodium chloride) or may be a separate solution containing different species (e.g., sodium sulphate). Depending on the stack configuration, anions and cations from the electrode stream may be transported into the concentrated stream, or *vice versa*. In each case, this transport is necessary to carry current across the stack and maintain electrically neutral stack solutions.

The performance of developed membranes was compared with the commercial membrane in terms of flux, current efficiency and energy consumption calculated by the following equations [43]:

(4) $$FLux=\frac{\Delta N}{At},$$

(5) $$\eta =\frac{Fz\Delta N}{n_{c}}\times 100,$$

(6) $$P=\frac{I\int^{\text{​}}Udt}{m},$$

where η = current efficiency of dilute (%), Δ*N* = $C_{d}^{n-1}V_{d}^{n-1}-C_{d}^{n}V_{d}^{n}\;$(mole), *C*_d_ = concentration of dilute (M), *V*_d_ = volume of dilute (L), *P* = energy consumption to produce water (KW·h·kg^−1^), *n*_c_ = number of cell pair (*n*_c_ = 1 in this case), *I* = direct current (A), *U* = applied potential (V), *m* = mass of removed salt (kg), and *t* = time (s).

#### 2.3.9. Desalination rate and Water Recovery Rate

The desalination rate *R* (%) was measured by Equation (7) [44]:

(7) $$R_{w}=\frac{\delta_{0}-\delta_{t}}{\delta_{t}}\times 100.$$

*R*_W_ is desalination rate (%), δ_o_ and δ_t_ (μm/cm) is a time of 0 min, and t min dilute compartment solution conductivity, respectively.

The water recovery rate was determined by Equation (8) [44]:

(8) $$\eta_{w}=\frac{V_{0}}{V_{t}}\times 100,$$

where η_w_ is water recovery rate (%); *V*_0_ and *V*_t_ is time of 0 min, and t min dilute compartment solution volume (mL).

## 3. Results and Discussion

### 3.1. FTIR Analysis

**Figure 2:**
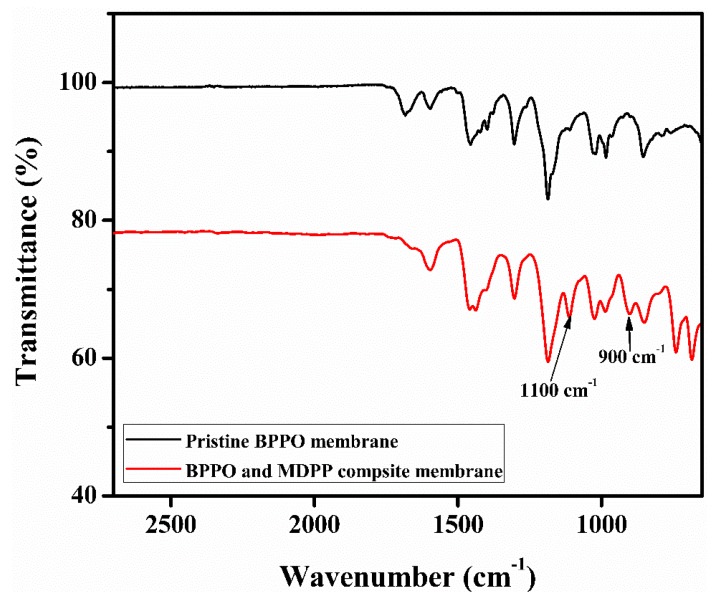
The IR spectrum of different anion exchange pristine BPPO membranes and BPPO and MDPP composite membranes.

To confirm the synthesis of anion exchange membranes from BPPO and MDPP, FTIR spectra of pristine and composite membranes are shown in Figure 2. The bands in the range of 1446 cm^−1^ are because of stretching of –CH groups [45]. The adsorption peaks for symmetrical and asymmetrical stretching vibration of C–O are at 1200 cm^−1^ and 1306 cm^−1^, and for phenyl groups are at 1470 cm^−1^ and 1600 cm^−1^. The attachment of quaternary phosphonium group in the composite membrane was confirmed by the appearance of two characteristic bands at 900 cm^−1^ and 1100 cm^−1^. While two sharp intense peaks around 756 cm^−1^ and 699 cm^−1^ indicated the presence of benzene rings in composite membranes [46]. These results support the reaction between BPPO and MDPP because, in pristine BPPO characteristics, peaks were absent.

### 3.2. Thermal Stability

**Figure 3:**
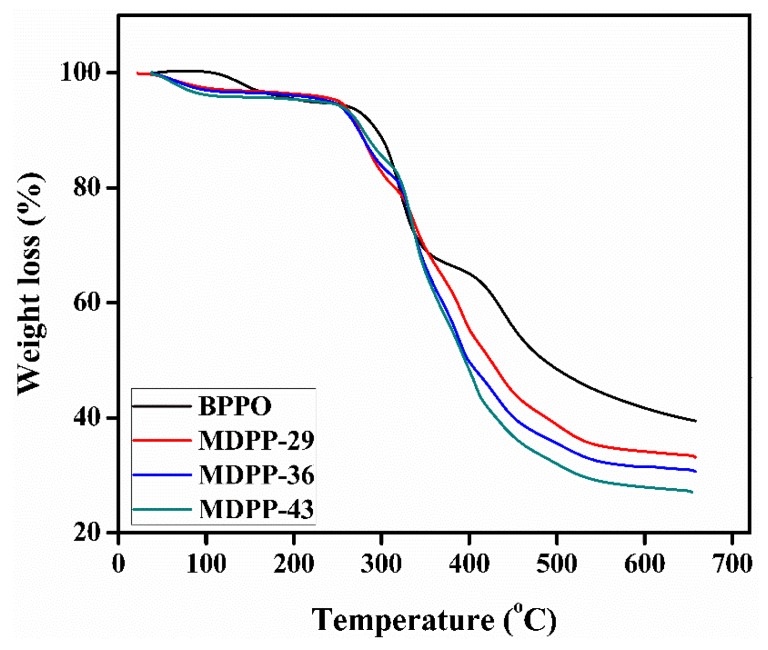
TGA thermograms for different membranes MDPP-0 to MDPP-43.

Thermal stability of prepared membranes was studied by TGA nitrogen atmosphere. The results are illustrated in Figure 3. The weight loss of the prepared membranes represented some significant steps, *i.e.*, water loss from the membrane phase, followed by dequaternization and finally the membrane degradation completed the whole process. The first weight loss occurred around 80–130 °C which confirmed the evaporation of residual water from the membrane matrix. The second degradation stage was noticed around 260–270 °C, which is due to the degradation of the quaternary phosphonium group. The final weight loss observed at 430 °C corresponds to the rest of polymer matrix.

### 3.3. Morphology of Membranes

**Figure 4:**
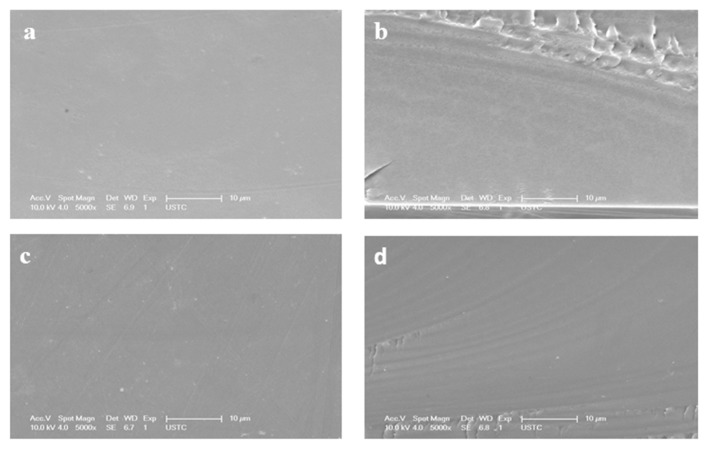
MDPP-36 membrane (**a**) surface area diagram (**b**) cross-section. MDP-43 membrane (**c**) surface area diagram (**d**) cross-section.

Figure 4 represents the SEM images of surface and cross-section of selected membranes. The prepared membranes are smooth and homogeneous, which confirms good compatibility between BPPO and MDPP contents. It is evident from the surface images of prepared membranes that the better miscibility can be obtained by improving MDPP contents. The absence of any holes, pores or cracks at prepared membranes signify homogeneity of membranes. The cross-section of the prepared anion exchange membranes is dense and compact, which can facilitate the electrodialysis process.

### 3.4. Water Uptake (W_R_), Ion Exchange Capacity and Linear Expansion Ratio (LER)

**Figure 5:**
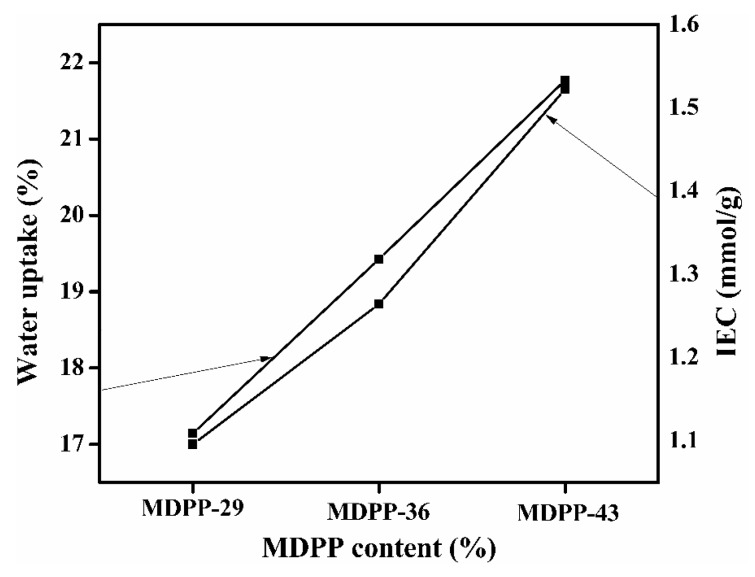
Ion exchange capacity and water uptake of membranes MDPP-29 to MDPP-43.

The charged nature of the prepared membranes was confirmed by IEC values via the titration method and shown in Figure 5. From the figure, it is clear that, the IEC value is 1.06–1.52 mmol/g, where the increase in value is directly associated with enriched MDPP contents in the membrane matrix. This increase in IEC is very important in terms of permeability and selectivity.

Figure 5 illustrates the water uptake by prepared ion exchange membrane. The W_R_ values are found to be increased with increasing ion exchange capacity of prepared membranes. Water uptake (W_R_) is regarded as a featured attribute of ion exchange membrane and has a significant effect on separation phenomena, dimensional as well as mechanical properties [47,48,49]. Water molecules in the membrane matrix facilitate the dissociation of charged functional groups and also very important for the transportation of ions [49].

Linear expansion ration (LER) of prepared membranes was studied in water and results are given in Table 1, *i.e.*, 7.96%–11.86%, and the value increases by increasing the ion exchange capacity and water uptake of membranes. This suggests that prepared membranes have good swelling resistance that further confirms their long lasting electrodialysis applications.

### 3.5. Mechanical Properties

Tensile strength (TS) demonstrates the membrane resistance to mechanical force while elongation at break (*E*_b_) represents the flexibility of membranes. The membranes with higher tensile strength always have a smaller elongation at break. The tensile strength and elongation at a break of prepared membranes are given in Table 2. The membrane MDPP-43 possesses the largest elongation at break and lowest tensile strength representing the higher flexibility of membranes. While the anion exchange membrane B shows the lowest elongation at break and highest tensile strength. The highest recorded *E*_b_ value is 647.98%, and the TS value is 30.77 MPa. It was observed that the TS values followed a decreasing trend and the *E*_b_ values followed an increasing trend. The tensile strength of prepared membranes varied from 16.66 to 30.77 MPa, which were higher than hybrid ion exchange membranes (7–16 MPa) [39], confirming that the membranes discussed were of good tensile strength (TS). The elongation at break (*E*_b_) varied from 485.57%–647.98%, which is much higher than the reported ones *i.e.*, 12.3%, [38] and 131%–187% [39], again depicting excellent flexibility of prepared membranes.

### 3.6. Membrane Area Resistance and Transport Number

Area resistance of an ion exchange membrane is a characteristic endowment to measure the energy employed in the electrodialysis process. Thus, ion exchange membranes with lower area resistance are selected for ED process. Area resistance of prepared membranes was calculated in 0.5 M NaCl as indicated in Table 3. The area resistance reduces with increasing MDPP contents and the ion exchange capacity of the membrane matrix. Out of three prepared membranes, two membranes (MDPP-36 and MDPP-43) have lower area resistance values compared with a commercial membrane Neosepta AMX.

In the electrodialysis process, the transport number of ion exchange membrane is explained as the current by the counter-ions. A higher transport number of membrane shows that lower amount of energy is employed in the ion-exchange phenomenon. The obtained transport numbers of prepared membranes are represented in Table 3. These transport numbers of membranes are found to be increased with the increase in MDPP content because the diffusion of co-ions across the membrane was suppressed with increasing quaternary ammonium group [50,51,52,53]. The membrane MDPP-43 shows the maximum value transport number (0.95) among all the anion exchange membranes. It could be related to the maximum suppression of co-ions diffusion by maximum quantity of quaternary phosphonium group in the membrane MDPP-43. Hence, these results suggested that the prepared anion exchange membranes have excellent capability toward chloride ions.

### 3.7. Membrane Performance

**Figure 6:**
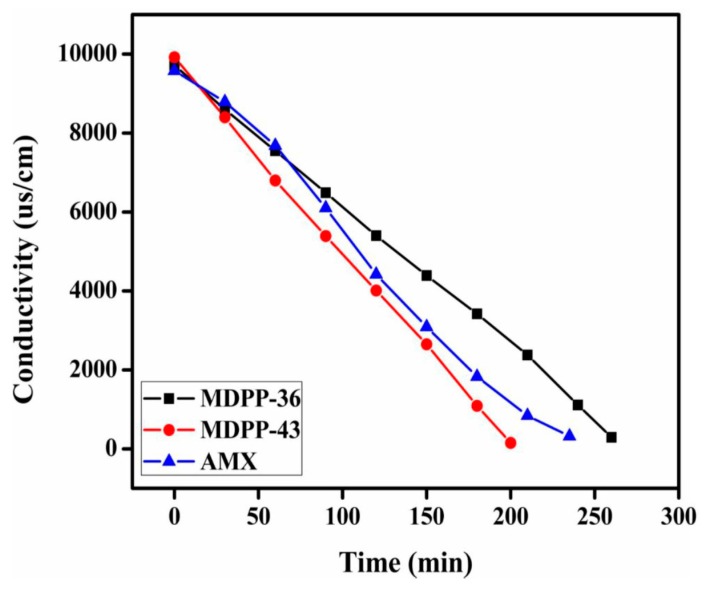
The change in conductivity in dilute cell during ED tests.

**Figure 7:**
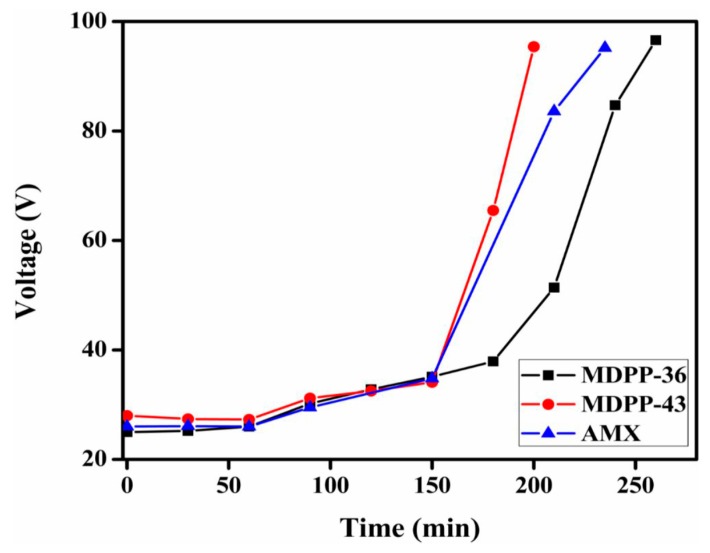
The change of potential over the stack with time.

After the complete characterization of prepared anion exchange membranes, the two membranes (MDPP-36 and MDPP-43) were tested for the removal of NaCl from its aqueous solution by electrodialysis, owing to their good mechanical properties. ED experiments were conducted by pumping feed solution (0.1 M NaCl) into dilute cells with flow rate of 60 mL/min and direct current of 28 mA/cm^2^. In dilute cells, the change in conductivity of NaCl with time is represented in Figure 6. It is clear from the figure that both of the membranes show decreases in conductivity with time, which may be due to the increased ion exchange content in the membrane matrix. Moreover, the potential over the stack for prepared membrane MDPP-43 is lower than the other two membranes investigated as shown in Figure 7, suggesting lower energy consumption. Membrane MDPP-43 showed better desalination performance than that of MDPP-36 and commercial membrane Neosepta AMX because Cl^−^ ions migrated rapidly from dilute cells to concentrated cells due to higher IEC and transport number values. Table 4 summarizes the desalination performance of the prepared membranes (MDPP-36 and MDPP-43) and commercial membrane Neosepta AMX. The results confirm that, under the same experimental conditions, the values of current efficiency, flux of salts and the energy consumption for prepared membrane MDPP-43 present better ED performance over the MDPP-36 as well as commercial membrane Neosepta AMX. Therefore, it can best be employed for salt removal from water.

To explain the desalination efficiency of studied membranes, the water recovery rate and desalination rate are given in Table 5. The prepared membranes have higher water recovery and desalination rate due to the dense morphology of fabricated membranes. Thus, the prepared membranes can be a good candidate for salt removal.

## 4. Conclusions

The anion exchange membranes were fabricated successfully from BPPO and MDPP for desalination via electrodialysis. This is a simple efficient process that could be the best alternative of highly carcinogenic and banned Chloromethyl ether (CME). The dense structure of membranes is confirmed by SEM study. The prepared membranes have higher thermal stability at elevated temperatures. These membranes have ion exchange capacity of 1.09 mmol/g to 1.52 mmol/g, water uptake of 17.14% to 21.77%, linear expansion ratio of 7.96% to 11.86%, tensile strength of 16.66 MPa to 30.77 MPa, elongation at break of 485.57% to 647.98%. These membranes are highly conductive and selective toward the Cl^−^. The anion exchange membrane showed excellent performance in removal of NaCl from water by electrodialysis. Therefore, the fabricated membranes (MDPP-36 and MDPP-43) could be efficient for ED desalination applications.

## Figures and Tables

**Table 1:** Composition, linear expansion ratio (LER) and thickness of membrane MDPP-29 to MDPP-43.

Membranes	MDPP-29	MDPP-36	MDPP-43	AMX
BPPO (g)	0.8	0.8	0.8	-
MDPP of BPPO (%)	29	36	43	-
LER (%)	7.96	8.67	11.86	7.65
Thickness (μm)	110	83	84	134 [44]

**Table 2:** Tensile strength and elongation at break of membranes MDPP-29 to MDPP-43.

Membranes	MDPP-29	MDPP-36	MDPP-43	AMX
TS (MPa)	30.77	23.97	16.66	40.59
*E*_b_ (%)	485.57	589.84	647.98	27.64

Where TS: Tensile strength, *E*_b_: Elonation at break.

**Table 3:** Area resistance and transport number of membrane MDPP-29 to MDPP-43.

Membranes	AMX	MDPP-29	MDPP-36	MDPP-43
Area resistance (Ω·cm^2^)	11.9	677	8.3	2.9
Transport number	0.99	0.92	0.94	0.95

**Table 4:** The membrane ED performance compared to commercial membrane Neosepta AMX.

Membranes	Operating Current (0.2 A)			
	Desalting Ratio (%)	Current Efficiency η (%)	Energy Consumption (*P*) (Kw·h·kg^−1^ water)	Flux (mole·m^−2^·h^−1^)
MPDD-36	84.6	45.2	41.45	4.82
MPDD-43	98.5	59.4	29.52	6.33
AMX	96.7	49.6	40.55	5.29

**Table 5:** Water recovery rate and desalination rate of studied membranes.

Membranes	Water Recovery Rate (%)	Desalination Rate (%)
MPDD-36	95	96.0
MPDD-43	98	98.49
AMX	97	97.0

## Abbreviations

The following abbreviations are used in this manuscript:

AEM	Anion exchange membrane
BPPO	Brominated poly(2,6-dimethyl-1,4-phenylene oxide)
MDPP	Methyl(diphenyl)phosphine
NMP	N-Methyl-2-pyrrolidone
W_R_	Water uptake
IEC	Ion exchange capacity
LER	Linear expension ration
AMX	Commercial anion exchange membrane
CMX	Commercial cation exchange membrane
*R*	Gas constant
*R*_W_	Desalination rate
SEM	Scanning electron microscopy
TS	Tensile strength
*E*_b_	Elongation at break
ED	Electrodialysis
